# Identification of Potentially Related Genes and Mechanisms Involved in Skeletal Muscle Atrophy Induced by Excessive Exercise in Zebrafish

**DOI:** 10.3390/biology10080761

**Published:** 2021-08-10

**Authors:** Chen-Chen Sun, Zuo-Qiong Zhou, Zhang-Lin Chen, Run-Kang Zhu, Dong Yang, Xi-Yang Peng, Lan Zheng, Chang-Fa Tang

**Affiliations:** Key Laboratory of Physical Fitness and Exercise Rehabilitation of the Hunan Province, College of Physical Education, Hunan Normal University, Changsha 410012, China; sunchenchen1022@hunnu.edu.cn (C.-C.S.); zhouzuoqiong26@aliyun.com (Z.-Q.Z.); zhanglinchen@hunnu.edu.cn (Z.-L.C.); zhurunkang@hunnu.edu.cn (R.-K.Z.); yangdong@hunnu.edu.cn (D.Y.)

**Keywords:** skeletal muscle atrophy, excessive exercise, FoxO signaling pathway, Wnt signaling pathway, p53 signaling pathway

## Abstract

**Simple Summary:**

Excessive exercise can lead to muscle atrophy, which is particularly concerning in professional athletes. However, the effects of excessive exercise on the skeletal muscle system remain unclear. Here, we used a zebrafish model of excessive exercise to identify genes that are dysregulated during excessive exercise. We mapped the identified genes to regulatory networks to gain an understanding of their functions during muscle atrophy. We identified several important mechanisms by which excessive exercise can lead to muscle wasting and prevent regeneration. Our findings provide fundamental knowledge on the effect of overtraining on the skeletal muscle system, and will enable better monitoring of muscle condition in athletes.

**Abstract:**

Long-term imbalance between fatigue and recovery may eventually lead to muscle weakness or even atrophy. We previously reported that excessive exercise induces pathological cardiac hypertrophy. However, the effect of excessive exercise on the skeletal muscles remains unclear. In the present study, we successfully established an excessive-exercise-induced skeletal muscle atrophy zebrafish model, with decreased muscle fiber size, critical swimming speed, and maximal oxygen consumption. High-throughput RNA-seq analysis identified differentially expressed genes in the model system compared with control zebrafish. Gene ontology and KEGG enrichment analysis revealed that the upregulated genes were enriched in autophagy, homeostasis, circadian rhythm, response to oxidative stress, apoptosis, the p53 signaling pathway, and the FoxO signaling pathway. Protein–protein interaction network analysis identified several hub genes, including keap1b, per3, ulk1b, socs2, esrp1, bcl2l1, hsp70, igf2r, mdm2, rab18a, col1a1a, fn1a, ppih, tpx2, uba5, nhlrc2, mcm4, tac1, b3gat3, and ddost, that correlate with the pathogenesis of skeletal muscle atrophy induced by excessive exercise. The underlying regulatory pathways and muscle-pressure-response-related genes identified in the present study will provide valuable insights for prescribing safe and accurate exercise programs for athletes and the supervision and clinical treatment of muscle atrophy induced by excessive exercise.

## 1. Introduction

Proper exercise can improve skeletal muscle adaptability through increased endurance and strength, by inducing mitochondrial regeneration, angiogenesis, and hypertrophy in skeletal muscle tissue [1,2]. In the process of adaptive formation, the recovery period after exercise is considered important to provide enough time for skeletal muscle metabolism and structural adaptation [3,4]. Therefore, the balance between muscle fatigue and recovery is particularly critical. Unfortunately, there is a lack of information regarding the appropriate recovery period of skeletal muscle for optimizing adaptation, causing frequent occurrence of unreasonable exercise [5]. About 60% of elite runners declared that they had suffered from overtraining syndrome [5]. Long-term imbalance between fatigue and recovery may eventually lead to muscle weakness, or even muscle atrophy, resulting in a diminished quality of life and an increase in both morbidity and mortality in numerous pathologies [6,7,8,9].

The most prominent feature of muscular atrophy is the loss of skeletal muscle, which is related to various physiological and pathological conditions, such as aging, trauma, immobilization, denervation, cancer, diabetes, inflammation, and starvation [10,11,12,13,14,15,16,17]. Skeletal muscle mass depends on the delicate equilibrium between the rates of protein synthesis and degradation [18]. Accumulating evidence suggests that the accumulation of protein misfolding and endoplasmic reticulum (ER) stress is caused by various catabolic stimuli, including aging, disease, and malnutrition [19,20,21], which may be a potential trigger of skeletal muscle atrophy. However, the molecular mechanisms of skeletal muscle atrophy induced by excessive exercise are unclear. Elucidating the pathogenesis of exercise-induced skeletal muscle atrophy is potentially helpful for controlling sports risk and providing new strategies for the treatment of muscular atrophy.

In zebrafish, skeletal muscle accounts for a large proportion of the trunk, and is highly similar to human muscle, both molecularly and histologically [22]. The relatively simple genome and easy gene manipulation of zebrafish are advantageous in myopathy and muscular dystrophy research [23,24,25]. Zebrafish are also an excellent model to facilitate excessive exercise interventions, because the aquatic environment is unlikely to cause injury following high-intensity forced exercise. In the present study, we trained zebrafish using a previously designed overtraining program and successfully constructed a model of excessive-exercise-induced skeletal muscle atrophy. Using this model, we applied high-throughput RNA-seq analysis to unravel the key genes and pathways that contribute to the pathogenesis of skeletal muscle atrophy induced by excessive exercise. Our findings contribute to the development of more biologically informed exercise regimens, particularly for professional sportspeople, which are less likely to cause injury by prescribing excessive exercise.

## 2. Materials and Methods

### 2.1. Animal Experiments

AB-strain male zebrafish (approximately 6 months old, *n* = 60) were purchased from the National Zebrafish Resource Center (Wuhan, China) and raised in a flow-through system with a water temperature of 28 ± 1 °C on a 12 h light/dark cycle. All animal experiments followed the Guidelines for Proper Conduct of Animal Experiments (Science Council of China) and were approved by the Ethics Committee of Hunan Normal University (approval number: 2018/046, approved date: 9 March 2019).

### 2.2. Swimming Exercises and Experimental Conditions

The zebrafish were randomly divided into two groups, both fed normally: the control group (*n* = 30) and the excessive exercise group (*n* = 30). The excessive exercise conditions were set up as described previously [26]. In brief, the excessive exercise group was forced to swim in a water current at a speed equivalent to 80% critical swimming speed (U_crit,_ 24 cm/s) for 4 weeks (6 h for 6 days per week). A month later, the zebrafish were sacrificed after deep anesthetization in 300 mg/L MS-222, and muscle tissue samples were collected and stored at −80 °C.

### 2.3. Hematoxylin and Eosin Staining

Hematoxylin and eosin (H&E) staining was performed as described previously [27]. The muscle tissues of zebrafish were fixed in 4% paraformaldehyde, dehydrated through an ethanol series, dewaxed with xylene, and embedded in paraffin wax. The samples were transversely cut into 4 μm thick slices, dewaxed, dried, and stained with hematoxylin and eosin under standard conditions. The images were captured with a microscope (Leica, Heidelberg, Germany) and analyzed using ImageJ (NIH, Bethesda, MD, USA).

### 2.4. Swimming Performance and Oxygen Consumption

Analysis of the swimming performance and oxygen consumption of zebrafish was performed using a miniature swimming tunnel respirator (Loligo Systems, Viborg, Denmark). The following formula was used to calculate U_crit_ values for the swimming tests: U_crit_ = Uf + US × (Tf/TS), where Uf (cm/s) is the highest velocity, US (2.7 L) is the velocity increment, Tf (min) is the time elapsed at fatigue velocity, and TS (14 min) is the prescribed interval time. U_crit_ is expressed in terms of body lengths per second (BL/s). Maximal oxygen consumption (MO_2_) was calculated using AutoRespTM 1 software (Loligo Systems, Viborg, Denmark).

### 2.5. Identification of Differentially Expressed Genes

Total RNA was extracted from the zebrafish skeletal muscle in the control and exercise groups, *n* = 3 for each group. Paired sequencing was performed using an Illumina NovaSeq 6000 platform (Illumina, San Diego, CA, USA) at Majorbio Technology Co., Ltd. (Shanghai, China). Trimmomatic (Max Planck Institute of Molecular Plant Physiology, Berlin, Germany) was used to process the acquired raw data, and pure data were obtained after discarding extended adaptor sequences and low-quality sequences. Hisat2 (University of Texas Southwestern Medical Center, Dallas, TX, USA) was used for gene mapping, and FeatureCounts was used for quantitative analysis. Comparison of the two groups was performed using the DESeq2 package to identify differentially expressed genes (DEGs).

### 2.6. Gene Ontology and Kyoto Encyclopedia of Genes and Genomes Enrichment Analysis of DEGs

To identify the physiological changes during skeletal muscle atrophy, the David online database (available online: https://david.ncifcrf.gov/ (accessed on 20 January 2021).) and ‘cluster profiler’ package in R were used to perform gene ontology (GO) and Kyoto encyclopedia of genes and genomes (KEGG) pathway enrichment analysis of DEGs.

### 2.7. Clusters of Orthologous Groups Analysis of DEGs

We used the bioMart package to obtain DEG protein sequences, and clusters of orthologous groups (COGs) analysis was performed using the COG database (NCBI, Bethesda, MD, USA).

### 2.8. Total RNA Extraction and qRT-PCR

The total RNA of zebrafish muscle tissue was extracted by homogenization in TRIzol solution according to the manufacturer′s protocol (Thermo Fisher Scientific, Waltham, MA, USA). RNA was converted to cDNA using a reverse transcription system kit (Takara, Tokyo, Japan). Real-time PCR was performed using a SYBR green master mix (Thermo Fisher Scientific, Waltham, MA, USA). Relative mRNA expression was determined using a Bio-Rad real-time PCR system (CFX96, Bio-Rad, Hercules, CA, USA). Sangon Biotech synthesized primers for the detected genes and the reference gene, *gapdh*. The relative mRNA expression was determined using the 2^−ΔΔCT^ method, and the primer sequences used are listed in Table S1.

### 2.9. Western Blot

The zebrafish skeletal muscle tissue was taken out and lysed in cold RIPA buffer (Solarbio, Beijing, China) containing a mixture of protease and phosphatase inhibitors (Solarbio, Beijing, China). Protein quantification was performed using a bicinchoninic acid (BCA) protein assay kit (Vazyme, E112-01/02, Nanjing, China), and the protein (30 mg) in each sample was separated by 10% or 15% SDS-PAGE and then transferred to 0.45 mm or 0.22 mm polyvinylidene difluoride (PVDF) membranes. The membranes of phosphorylated protein were blocked with 5% BSA, and others were blocked with 5% fat-free milk. Membranes were incubated with the primary antibodies overnight at 4 °C and washed with TBST. Subsequently, membranes were incubated with appropriate secondary HRP-linked antibodies. The proteins were detected with a gel imaging system (Tanon, Shanghai, China). The antibodies used in the present research were as follows: rabbit anti-GAPDH antibody (1:2000, Servicebio, Wuhan, China), rabbit anti-MURF antibody (1:2000, Abcam, Boston, MA, USA), rabbit anti-FBXO32 antibody (1:2000, Abcam, Boston, MA, USA), rabbit anti-P53 antibody (1:2000, Abcam, Boston, MA, USA), rabbit anti-AKT antibody (1:2000, CST, Boston, MA, USA), rabbit anti-p-AKT antibody (1:2000, CST, Boston, MA, USA), rabbit anti-PI3K antibody (1:1000, Proteintech, Wuhan, China), rabbit anti-p-PI3K antibody (1:2000, CST, Boston, MA, USA), rabbit anti-LC3 antibody (1:1000, Proteintech, Wuhan, China), rabbit anti-BCL-2 antibody (1:1000, 1:1000, Proteintech, Wuhan, China), and rabbit anti-AMPK antibody (1:1000, 1:1000, Proteintech, Wuhan, China). The expressions of the phosphorylated proteins p-AKT and p-PI3K were normalized to total AKT and PI3K, and the expressions of other proteins were normalized to GAPDH.

### 2.10. Protein–Protein Interaction Network

The protein–protein interaction (PPI) network was predicted using the ‘search tool for the retrieval of interacting genes’ (STRING) online database. Interactions with a combined score > 0.7 were considered statistically significant. The bioinformatics platform Cytoscape was used to visualize the molecular interaction network, and the MCODE extension was used to cluster the PPI network of DEGs.

### 2.11. Statistical Analysis

Statistical analysis was performed using SPSS (version 22.0; IBM, Chicago, IL, USA) or GraphPad Prism software (version 7.0; San Diego, CA, USA). Data were expressed as the mean ± SD of three independent experiments. Unpaired *t*-tests were used to compare the mean values of the two groups. All experiments were repeated three times. Statistical significance was set at *p* < 0.05.

## 3. Results

### 3.1. Excessive Exercise Caused Muscle Atrophy of Zebrafish Skeletal Muscle

**Figure 1:**
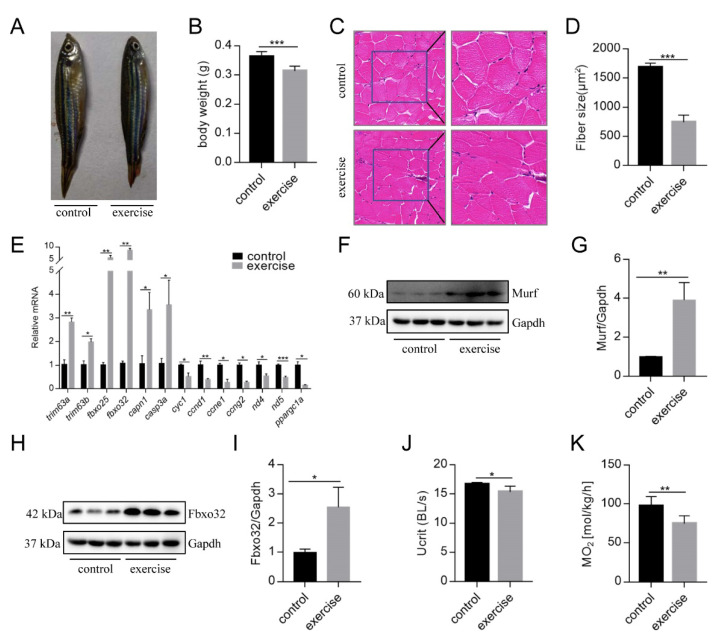
Excessive exercise causes skeletal muscle atrophy in zebrafish. (**A**) Representative image of excessive exercise and control zebrafish. (**B**) Changes in the body weight of zebrafish with or without excessive exercise over a 4-week period. (**C**) Representative photomicrographs of muscle sections stained with H&E. Scale bars = 20 μm. (**D**) Average fiber size (based on H&E staining). (**E**) The expression of muscle-atrophy-related genes (*trim63a*, *trim63b*, *fbxo25*, *fbxo32*, *capn1,* and *casp3a*), cyclin (*ccnd1*, *ccne1,* and *ccng2*), mitochondrial function markers (*cyc1*, *nd4*, and *nd5*), and *ppargc1a* in muscle tissue detected using real-time PCR. (**F**–**I**) Western blot analysis of Murf and Fbox32 proteins (representative blot). All uncut western blot images are provided in Supplementary Materials. Data represent mean ± SD (*n* = 6). (**J**,**K**) The critical swimming speed (U_crit_) and oxygen consumption (MO_2_) were measured using the Loligo System. * *p* < 0.05, ** *p* < 0.01, and *** *p* < 0.001.

After 4 weeks of excessive exercise, we found that the body size of the zebrafish in the excessive exercise group was significantly smaller than that of the control zebrafish (Figure 1A,B). H&E staining showed that the muscle fiber size of transverse sections in the excessive exercise group was 55.7% smaller than that in the control group (Figure 1C,D). The expression levels of the muscle atrophy marker genes *trim63a*, *trim63b*, *fbxo25*, *fbxo32*, *capn1,* and *casp3a* increased in the excessive exercise group compared with the control group (Figure 1E). Simultaneously, the expression levels of cyclin-encoding genes (*ccnd1, ccne1, ccng2*), mitochondrial cytochrome C protein (*cyc1*), NADH dehydrogenase 4 (*nd4*), NADH dehydrogenase 5 (*nd5*), and nuclear transcription coactivated peroxisome (*ppargc1a*) decreased compared with the control group (Figure 1E). We also found that the protein expressions of muscle atrophy markers (Murf and Fbox32) were evidently upregulated in the excessive exercise group compared with the control group (Figure 1F–I). Next, to study the changes in zebrafish exercise capacity under excessive exercise, we compared U_crit_ and MO_2_ in the excessive exercise and control zebrafish. Excessive exercise reduced the U_crit_ and MO_2_ of the zebrafish, suggesting decreased athletic ability as a result of excessive exercise (Figure 1J,K). Overall, these data indicate that excessive exercise causes muscle atrophy in zebrafish skeletal muscle.

### 3.2. Functional Annotation of DEGs

**Figure 2:**
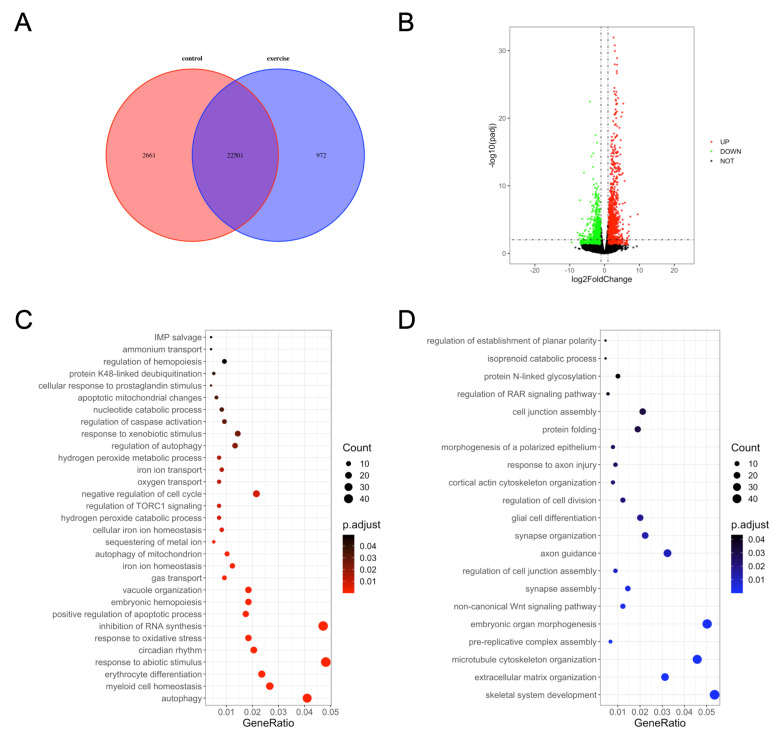
Gene ontology (GO) enrichment analysis of differentially expressed genes in zebrafish skeletal muscles. (**A**) Venn diagram of the total number of identified genes. (**B**) Volcano plot constructed using fold change values and *Padj* value. Red: upregulated, green: downregulated, gray: nonsignificant. (**C**) GO terms enriched in upregulated genes. (**D**) GO terms enriched in downregulated genes.

We next investigated the underlying mechanisms of excessive-exercise-induced skeletal muscle atrophy using high-throughput transcriptome analysis. Compared with the control group, 972 genes (3.7%) were specifically expressed and 2661 genes (10.2%) were not detected in the exercise-atrophied zebrafish skeletal muscle (Figure 2A). In the paired control and skeletal muscle atrophy samples, 2499 mRNA transcripts were differentially expressed between the groups (*Padj* < 0.05, fold changes > 2). Among these, 1337 were upregulated and 1162 were downregulated as a result of excessive exercise (Figure 2B). GO enrichment analysis showed that the enriched terms for the upregulated genes included autophagy, myeloid cell homeostasis, erythrocyte differentiation, response to abiotic stimulus, circadian rhythm, positive regulation of the apoptotic process, regulation of caspase activation, negative regulation of cell cycle, regulation of TORC1 signaling, inhibition of RNA synthesis, and response to oxidative stress (Figure 2C, Table S2). Conversely, the downregulated genes were significantly enriched in skeletal system development, extracellular matrix (ECM) organization, microtubule cytoskeleton organization, prereplicative complex assembly, noncanonical Wnt signaling pathway synapse assembly, axon guidance, cell junction assembly, and protein folding (Figure 2D, Table S3).

### 3.3. KEGG Pathway and COG Analysis of DEGs

**Figure 3:**
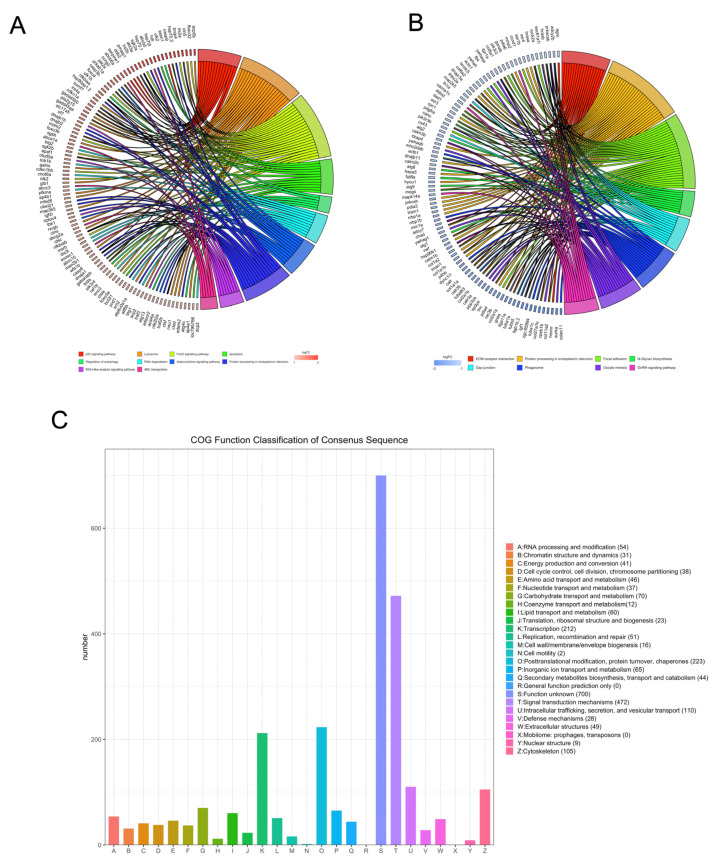
Kyoto encyclopedia of genes and genomes and clusters of orthologous groups (COGs) analysis of DEGs. The David online database and ‘clusterProfiler’ package in R were used to analyze the gene ontology (GO) terms associated with upregulated DEGs (**A**) and the GO terms associated with downregulated DEGs (**B**). (**C**) The BioMart package and COG database were used to obtain the COG assignments of the DEGs. The vertical axis represents the number of DEGs in each category, and the horizontal axis represents the COG functional category.

The DEGs were mapped to known pathways by KEGG pathway analysis, and most of the upregulated genes were enriched in the p53 signaling pathway, lysosomes, FoxO signaling pathway, apoptosis, regulation of autophagy, RNA degradation, adipocytokine signaling pathway, protein processing in the ER, RIG-I-like receptor signaling pathway, and ABC transporters (Figure 3A). The downregulated genes were enriched in ECM–receptor interaction, protein processing in the ER, focal adhesion, N-glycan biosynthesis, gap junction, phagosomes, oocyte meiosis, and GnRH signaling pathway (Figure 3B). We also classified the DEGs according to the homology group database in the COG database. Signal transduction mechanism, transcription, posttranslational modification, and cytoskeleton were the most representative functional clusters among the DEGs (Figure 3C).

**Figure 4:**
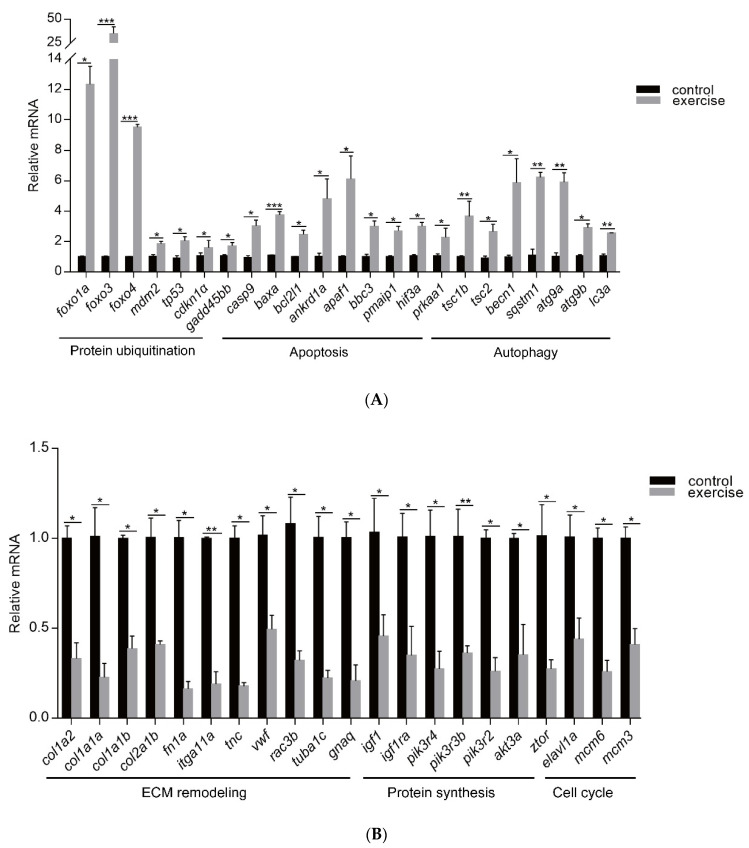
qRT-PCR was used to quantify key genes related to muscle atrophy caused by excessive exercise. (**A**) Upregulated genes. (**B**) Downregulated genes. The data represent mean ± SD (*n* = 6). * *p* < 0.05, ** *p* < 0.01, and *** *p* < 0.001.

**Figure 5:**
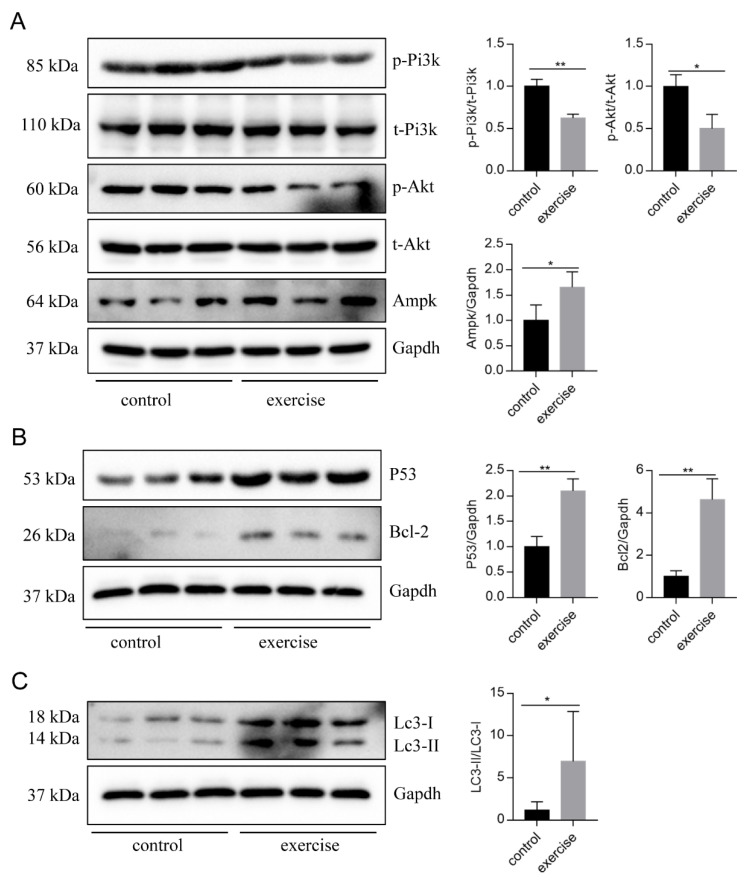
Western blot was used to validate key proteins in signals involved in excessive-exercise-induced muscle atrophy. (**A**) The expression of proteins in the PI3K–AKT signal (representative blot). (**B**) The expression of proteins in the apoptosis signal (representative blot). (**C**) The expression of proteins in the autophagy signal (representative blot). Right panels are quantitative analysis data of the protein expression level by Image J and GraphPad Prism. All uncut Western blot images are provided in Supplementary Materials. Data represent mean ± SD (*n* = 6). * *p* < 0.05, ** *p* < 0.01.

Next, to identify the high-level GO terms that explain the pathology of muscle atrophy after excessive exercise, we validated the expression of genes enriched in these GO terms by RT-qPCR. We found that genes involved in protein ubiquitination, including the FoxO signaling pathway (foxo1a, foxo3, foxo4, and mdm2), p53 signaling pathway (tp53, cdkn1a, and gadd45bb), apoptosis (casp9, baxa, bcl2l1, ankrd1a, apaf1, bbc3, pmaip1, and hif3α), and autophagy (prkaa1, tsc1b, tsc2, becn1, sqstm1, atg9a, atg9b, and lc3a) were upregulated after excessive exercise (Figure 4A). Genes involved in ECM remodeling, including ECM–receptor interaction (col1a2, col1a1a, col1a1b, col2a1b, and fn1a), focal adhesion (itga11a, tnc, vwf, and rac3b), gap junctions (tuba1c and gnaq), protein synthesis (igf1, igf1ra, pik3r4, pik3r3b, pik3r2, akt3a, and ztor), and cell cycle (elavl1a, mcm6, and mcm3) were downregulated (Figure 4B). Further, we validated the expression of the PI3K–AKT signal, the key signal of protein synthesis, and found that the phosphorylation levels of Pi3k and Akt were downregulated, and the expression of Ampk was upregulated (Figure 5A). We also verified the expression of proteins in the apoptosis signal (p53 and Bcl2) and autophagy signal (Lc3) and found that the expressions of these proteins in the excessive exercise group were upregulated compared with the control group (Figure 5B,C).

### 3.4. Construction of PPI Network and Cluster Identification

**Figure 6:**
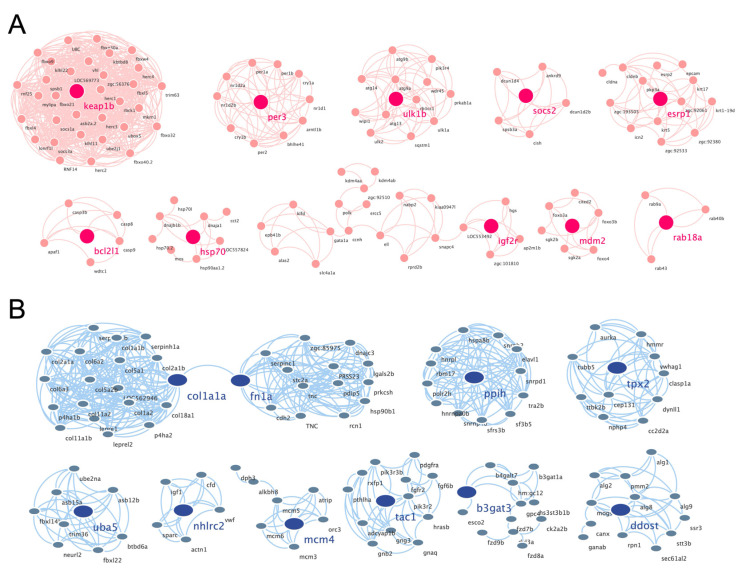
PPI networks of the cluster proteins relating to muscle atrophy caused by excessive exercise. The STRING online database and Cytoscape bioinformatics platform were used to analyze the upregulated (**A**) and downregulated DEGs (**B**) (*p* < 0.05, fold change > 2).

The PPI networks of the upregulated and downregulated genes were generated using the STRING database and Cytoscape software (Figure S1A,B), and the top 10 clusters in the PPI networks were generated using the MCODE extension (Figure 6A,B). The genes keap1b, per3, ulk1b, socs2, esrp1, bcl2l1, hsp70, igf2r, mdm2, and rab18a were the hub genes in the clusters of the PPI network of upregulated genes with the highest fold change. Similarly, col1a1a, fn1a, ppih, tpx2, uba5, nhlrc2, mcm4, tac1, b3gat3, and ddost were the hub genes in the clusters of the PPI network of downregulated genes (Table 1). Finally, we performed an additional round of GO enrichment analysis on the proteins in the PPI network. The 10 representative biological process GO terms are listed in Table 2. We found that most of the upregulated genes were involved in protein ubiquitination, circadian regulation of gene expression, response to abiotic stimulus, response to hydrogen peroxide, erythrocyte homeostasis, ADP metabolic process, negative regulation of the biosynthetic process, embryonic hemopoiesis, proteasome-mediated ubiquitin-dependent protein catabolic process, and autophagy. The most downregulated genes were involved in the ECM organization, skeletal system development, axon guidance, regulation of the retinoic acid receptor signaling pathway, noncanonical Wnt signaling pathway, glycoprotein biosynthetic process, carbohydrate derivative biosynthetic process, retinoic acid metabolic process, glial cell differentiation, and cellular hormone metabolic process.

## 4. Discussion

**Figure 7:**
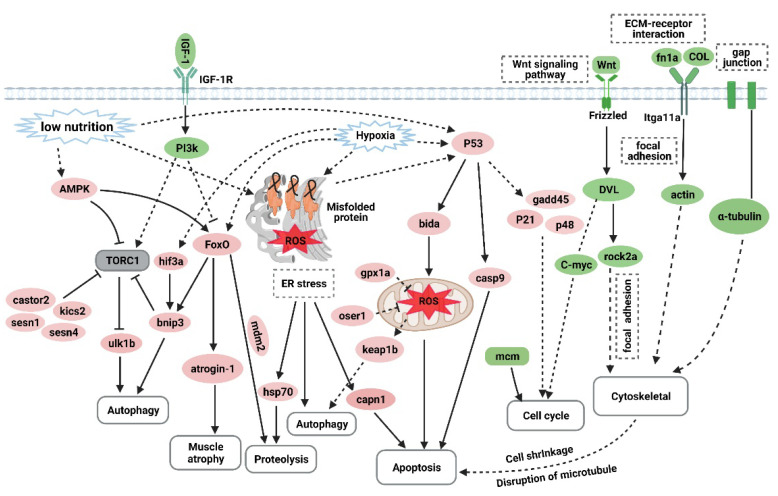
The hypothetical pathogenesis mechanism of muscle atrophy induced by excessive exercise in zebrafish. The upregulated genes are shown in red ovals, and the downregulated genes are shown in green ovals. In response to excessive exercise, the muscle cells may undergo the following events: (1) the inhibition of IGF–PI3K signaling and upregulation of AMPK inhibit the function of the TORC1 complex to suppress protein synthesis and promote autophagy; (2) excessive exercise activates FoxO signaling, which promotes the expression of protein hydrolysis, autophagy, and muscle-atrophy-related gene expression; (3) excessive exercise induces oxidative stress, and the accumulation of misfolded proteins in the endoplasmic reticulum and the activation of p53 signaling lead to proteolysis, autophagy, and apoptosis; (4) overtraining inhibits Wnt signaling and the expression of the MCM complex, which suppresses the proliferation of muscle satellites; and (5) excessive exercise destroys ECM–receptor interaction, focal adhesion, and the gap junction of muscle cells, leading to cytoskeleton damage and inducing apoptosis. (Created with BioRender.com.)

Overtraining is a potential cause of muscle atrophy [7]. After applying an overtraining regimen in zebrafish to model excessive exercise, the morphology of zebrafish skeletal muscles displayed decreased mass and cross-sectional muscle fiber size. The U_crit_ and MO_2_ of zebrafish in the excessive exercise model also decreased significantly compared with the control group. Based on these factors, we successfully established a zebrafish model of exercise-induced muscle atrophy that presented a measurable decline in exercise performance. Transcriptome analysis of the skeletal muscle of zebrafish under excessive exercise revealed that overtraining could lead to changes in protein synthesis and degradation, redox homeostasis, ER stress and apoptosis, muscle regeneration, and ECM remodeling, which may contribute to the pathological mechanism of excessive-exercise-induced muscle atrophy. A schematic regulatory network for excessive-exercise-induced muscle atrophy in zebrafish is presented in Figure 7.

Skeletal muscle mass depends on the balance between the rate of protein synthesis and degradation. Only when the rate of protein synthesis exceeds the rate of degradation will the muscle mass increase; otherwise, the muscle will decrease in mass, or even atrophy may occur in severe cases [28]. Protein synthesis depends on intracellular mTOR signaling, which is activated by insulin/IGF-1–PI3K–Akt cascade phosphorylation signals [29,30]. The genetic inactivation of IGF1R or mTOR in the muscle can result in decreased muscle fiber size or muscular dysplasia [31,32]. However, in the excessive exercise zebrafish model, the PI3K–AKT signal igf1, igf1ra, pik3r4, pik3r3b, pik3r2, and akt3a were significantly downregulated. Importantly, the phosphorylation levels of Pi3k and Akt proteins were downregulated. The downregulation of PI3K–AKT phosphorylation signals may contribute to inhibited protein synthesis after excessive exercise training. In contrast, members of the FoxO family, such as foxO1, foxO3, and foxO4, as negative regulators of insulin/IGF-1 signaling, were significantly upregulated. FoxO signaling, which is an important intracellular emergency response to starvation or hypoxia, can cause protein degradation through the ubiquitin–proteasome and autophagy–lysosome pathways [33]. Consistently, the GO analysis of upregulated genes showed that autophagy was the most significantly enriched term. The transcriptional targets of FoxO proteins, such as atrogin-1 (fbox32) and MuRF-1 (trim63a, trim63b), which mediate the ubiquitination and subsequent degradation of muscle structural proteins [33,34], were also significantly upregulated. The inhibition of mTOR signaling and the activation of FoxO signaling suggest that the total amount of protein in zebrafish skeletal muscles is reduced after excessive training. Under normal conditions, autophagy can maintain cell homeostasis by clearing metabolic waste. Autophagy is a mechanism of maintaining survival under external pressure, hypoxia, starvation, and ER stress. According to the transcriptional analysis, the protein-folding-related genes in the ER were downregulated, while oxidative stress response genes were upregulated, indicating that ER stress and oxidative stress also cause autophagy. Reactive oxygen species (ROS) are metabolites of oxygen consumption and important signaling molecules in muscles that maintain increased contractile activity [35]. However, excessive ROS levels that exceed the abilities of antioxidant defenses and disturb redox homeostasis negatively affect muscle contractile proteins, mitochondrial phospholipids, and DNA, and are associated with the pathophysiology of muscle aging (sarcopenia) and various muscular disorders [36,37,38,39]. The mitochondria are the main sites of ROS production. Oxidative stress can trigger mitochondrial autophagy to recover nutrients and reduce ROS. In the excessive exercise zebrafish model, the upregulated genes were significantly enriched in mitochondrial autophagy and oxidative stress. In addition to the mitochondria, the ER of skeletal muscle cells also produces ROS to trigger muscle contraction. Recent evidence suggests that ER stress is associated with skeletal muscle atrophy caused by aging, disease, and malnutrition [40,41]. Many stimuli, such as exercise, hypoxia, redox balance changes, and nutrition/energy deprivation, can interfere with the homeostasis of the ER, which may lead to the accumulation of unfolded and misfolded proteins in the ER lumen, eventually resulting in ER stress and autophagy to maintain protein homeostasis by scavenging nonfunctional proteins. In addition, ER stress can activate p53 signaling and induce apoptosis [42,43,44,45]. In our excessive exercise zebrafish model, p53 signaling and apoptosis were significantly enriched, and the RT-qPCR showed that p53-signaling-pathway-related genes, such as tp53, cdkn1α, and gadd45bb, and apoptosis-related genes, such as lc3a, baxa, bcl2l1, ankrd1a, and casp9, were upregulated. Although p53 signaling can respond to cell pressure and regulate autophagy, apoptosis, and the cell cycle to maintain cell homeostasis, dysfunctional p53 signaling is associated with a variety of diseases. Increased expression of p53 and its target genes has been previously observed in muscle atrophy models of aging [46], muscle denervation [47], and Huntington′s disease [48]. Therefore, we speculate that the starvation and oxidative stress induced by excessive exercise lead to ER pressure and activate p53 signaling. Autophagy and apoptosis induced by excessive exercise are two of the causes of exercise-induced muscular atrophy.

In addition to the homeostasis of muscle cells, the strong regenerative ability of muscle satellite cells also plays a key role in the maintenance of adult muscle tissue homeostasis. Stimulated by growth signals or physical trauma, activated satellite cells proliferate and differentiate into mature myocytes, replacing lost or senescent myocytes. In a mouse model with severe muscle injury, the proliferation of stem cells could completely regenerate muscles, and normal exercise could be resumed after severe muscle injury [49,50]. Unfortunately, in the present study, the cell cycle arrest genes cdkn1a, cdkn3, and cdkn1bb were upregulated. According to the GO enrichment analysis of the DEGs, the Wnt signaling pathway, which has been implicated in muscle repair and regulates satellite cell differentiation and self-renewal, was inhibited, potentially affecting myocyte regeneration. The suppression of the cell cycle and Wnt signaling suggests that weakened muscle regenerative capabilities also contribute to skeletal muscle atrophy induced by excessive exercise.

Transcriptional analysis showed that ECM–receptor interactions and the focal adhesion of overexercised zebrafish skeletal muscle were also repressed. The ECM plays a key role in maintaining the quality and strength of skeletal muscles [51]. Degradation of the ECM leads to muscle damage caused by structural and functional changes to myocytes [52]. The loss of collagen and fibroblast function, and the increase in matrix metalloproteinase activity, can promote the degradation of the ECM, leading to cell shrinkage and senescence [53]. qRT-PCR verified that ECM (col1a2, col1a1a, col1a1b, col2a1b, and fn1a), focal adhesion (itga11a, vwf, and rac3b), and gap junction (tuba1c and gnaq) genes were downregulated by excessive exercise, indicating that the ECM of the skeletal muscle was degraded. However, whether this indicates a phenotype or the cause of muscle atrophy needs further study.

Although nerve loss is the main characteristic of spinal muscular atrophy, we found that some key factors of axon guidance were significantly downregulated by overtraining in zebrafish. The key to repairing defects of the peripheral nervous system is to promote the regeneration of axons and prevent the atrophy of muscles under the control of the nervous system [54]. During such repair, axon guidance molecules play an important role in the orderly growth of axons [55]. In the present study, the downregulation of key factors relating to axon guidance indicates that axons may be unable to form in an orderly manner during or after excessive exercise, further contributing to muscle atrophy. Further research and analysis of these axon guidance factors could enable the prevention and treatment of muscle atrophy caused by peripheral nervous system defects.

## 5. Conclusions

Through the construction of an excessive exercise zebrafish model and high-throughput sequencing analysis, we uncovered the potential mechanisms of excessive-exercise-induced skeletal muscle atrophy. However, the order of these events has not been clearly unraveled. Further, detailed exercise intervention regimes and sampling analysis will provide more information about the order of these events during muscle atrophy. As zebrafish and human muscles are extremely similar, this model could be used for testing newly developed drugs targeting muscle atrophy. Furthermore, the underlying regulatory pathways and hub genes that correlate with the pathogenesis of skeletal muscle atrophy induced by excessive exercise can be used to facilitate informed clinical decision making and to prescribe safe training regimens for athletes. Taken together, these pathways will provide valuable insights for the supervision and clinical treatment of muscle atrophy induced by excessive exercise.

## Figures and Tables

**Table 1:** Hub genes of clusters of protein–protein interaction networks.

	Hub Gene	Description
UP	keap1b	kelch-like ECH-associated protein 1b
	per3	period circadian clock 3
	ulk1b	unc-51-like kinase 1b
	socs2	suppressor of cytokine signaling 2
	esrp1	epithelial splicing regulatory protein 1
	bcl2l1	bcl2-like 1
	hsp70	heat shock cognate 70-kd protein
	igf2r	insulin-like growth factor 2 receptor
	mdm2	MDM2 oncogene, E3 ubiquitin protein ligase
	rab18a	RAB18A, member RAS oncogene family
DOWN	col1a1a	collagen, typeI, alpha1a
	fn1a	fibronectin 1a
	ppih	peptidylprolyl isomerase h
	tpx2	tpx2, microtubule-associated, homolog
	uba5	ubiquitin-like modifier activating enzyme 5
	nhlrc2	nhl repeat containing 2
	mcm4	minichromosome maintenance complex component 4
	tac1	tachykinin 1
	b3gat3	beta-1,3-glucuronyltransferase 3
	ddost	dolichyl-diphosphooligosaccharide-protein glycosyltransferase subunit

**Table 2:** Gene ontology enrichment analysis of the intersect proteins of gene clusters.

	ID	Term	*p*-Value
Up	GO:0016567	protein ubiquitination	8.60 × 10^−14^
	GO:0032922	circadian regulation of gene expression	9.17 × 10^−12^
	GO:0009628	response to abiotic stimulus	9.19 × 10^−8^
	GO:0042542	response to hydrogen peroxide	2.22 × 10^−6^
	GO:0034101	erythrocyte homeostasis	1.089 × 10^−5^
	GO:0046031	ADP metabolic process	1.34 × 10^−5^
	GO:0009890	negative regulation of biosynthetic process	1.76 × 10^−5^
	GO:0035162	embryonic hemopoiesis	3.25 × 10^−5^
	GO:0043161	proteasomal pathway	3.61 × 10^−5^
	GO:0006914	autophagy	4.51 × 10^−5^
Down	GO:0030198	extracellular matrix organization	2.00 × 10^−7^
	GO:0001501	skeletal system development	4.01 × 10^−5^
	GO:0007411	axon guidance	0.00045
	GO:0048385	regulation of retinoic acid receptor signaling pathway	0.00051
	GO:0035567	noncanonical Wnt signaling pathway	0.00054
	GO:0009101	glycoprotein biosynthetic process	0.0013
	GO:1901137	carbohydrate derivative biosynthetic process	0.0014
	GO:0042573	retinoic acid metabolic process	0.0015
	GO:0010001	glial cell differentiation	0.0016
	GO:0034754	cellular hormone metabolic process	0.0020

## Data Availability

The data used to support the findings of this study are available from the corresponding author upon request.

## Supplementary Materials

The following are available online at https://www.mdpi.com/article/10.3390/biology10080761/s1: Figure S1: The up-regulated genes were generated using the STRING database and Cytoscape software, Table S1: List of primers used for RT-qPCR, Table S2: The GO analysis of up-regulated genes, Table S3: The GO analysis of down-regulated genes, Figure S1A: The PPI network of up-regulated genes, Figure S1B: The PPI network of down-regulated genes, File S1: Western blot original images.
