# Supplementary material for: Identification of Potentially Related Genes and Mechanisms Involved in Skeletal Muscle Atrophy Induced by Excessive Exercise in Zebrafish
Source: Biology (Basel). 2021 Aug 10;10(8):761. doi: 10.3390/biology10080761 (PMC8389602; doi:10.3390/biology10080761)

Supplementary Figure S1a. The up-regulated genes were generated using the STRING database and Cytoscape software.

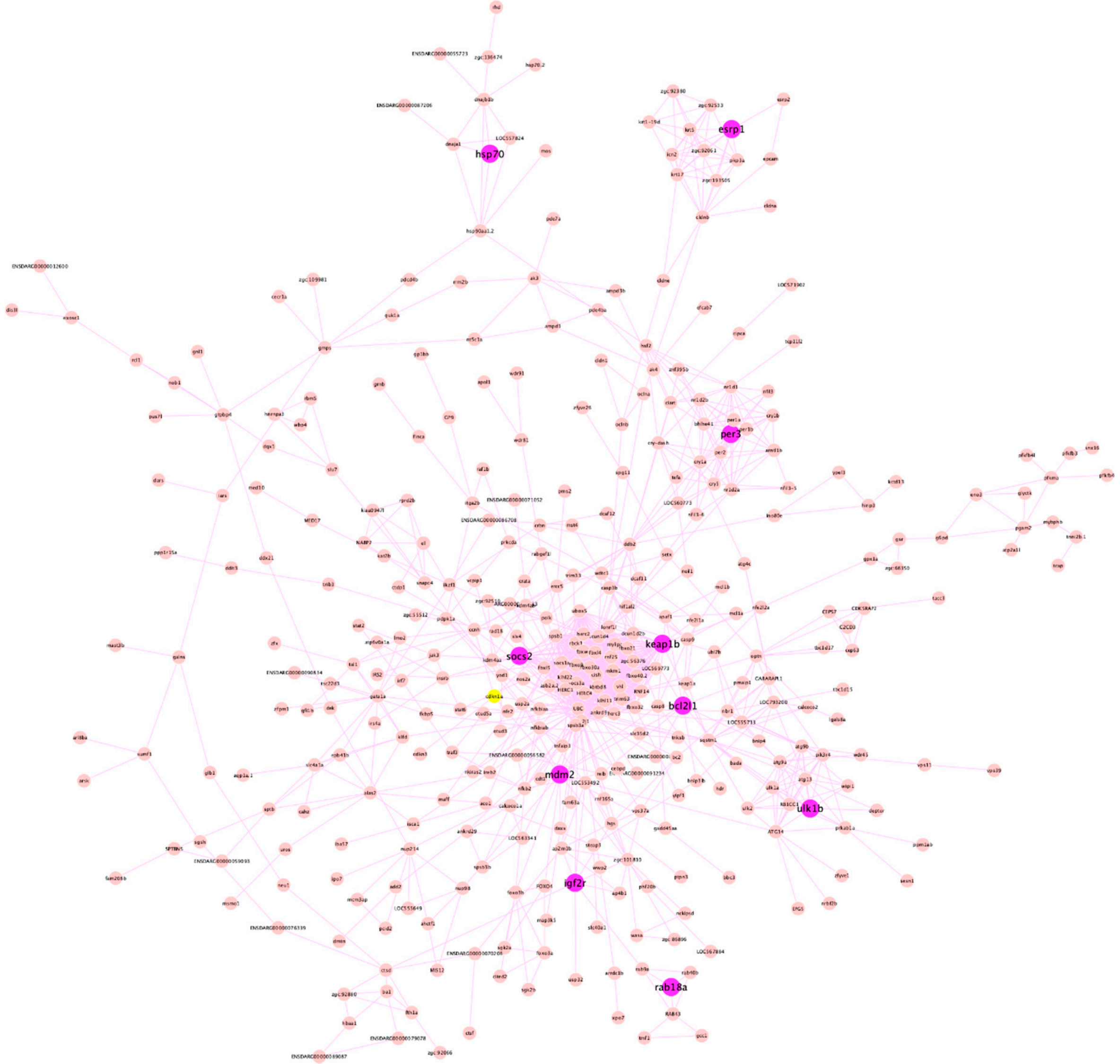

Supplementary Figure S1b. The down-regulated genes were generated using the STRING database and Cytoscape software.

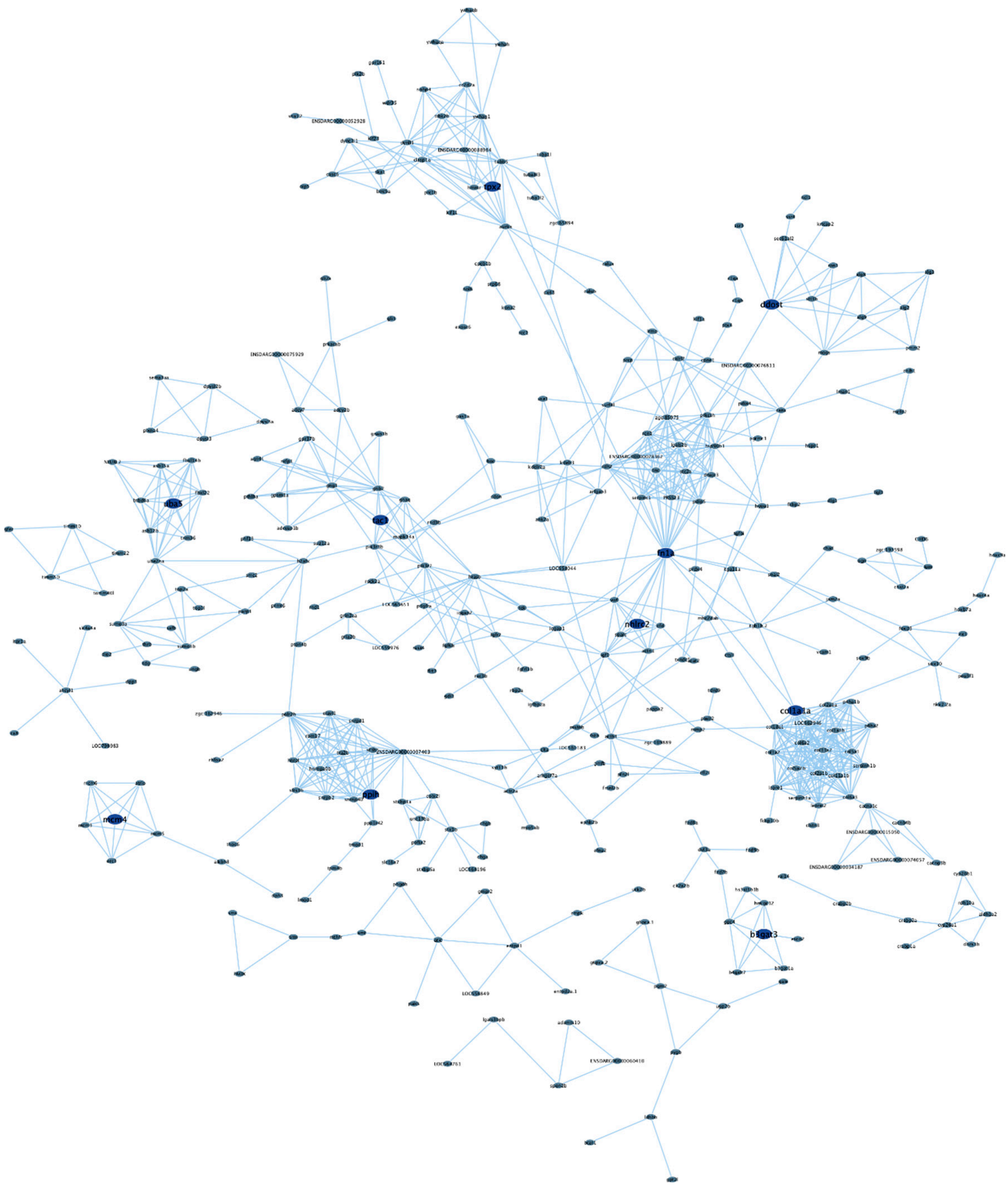

Supplement: Supplementary file 1 [file biology-10-00761-s001.zip › Supplementary figure1A-B.pdf]
