# Supplementary material for: Identification of Potentially Related Genes and Mechanisms Involved in Skeletal Muscle Atrophy Induced by Excessive Exercise in Zebrafish
Source: Biology (Basel). 2021 Aug 10;10(8):761. doi: 10.3390/biology10080761 (PMC8389602; doi:10.3390/biology10080761)
Supplement: Supplementary file 1 [file biology-10-00761-s001.zip › Supplementary Table 1.pdf]

Supplementary Table S1. List of primers used for RT-qPCR

Gene	Accession number	Forward primer (5′–3′)	Reverse primer (5′–3′)
ankrd1a	XM_001920057.5	AATTCAACTCCAGAGACAAGC	CGTACATCATCAGCAGTCTAA
apaf1	NM_131608.1	TCCTATGACTATGAAGCACTG	ACAGAACAGACAGAACCTTAG
atg9a	NM_001083031.1	ATGACATCCTCTTCGCCAACAA	ACACTGTCACGGATCCTAGC
atg9b	NM_001320078.1	TTCACTACATGCCCGACCAC	CCTCCAAGATGAAGACCGCT
baxa	NM_131562.2	GGCTATTTCAACCAGGGTTCC	TGCGAATCACCAATGCTGT
bcl2l1	NM_131807.1	CATTGGACTTACAGAAGACAC	CTAGCGTTCGTTCTATTCATG
bbc3	NM_001045472.2	GAGATGAACGCTGTCTTCCTT	GTAGAGGGCATTGATGGTGTC
becn1	NM_200872.1	GCAGGTAAGTTTGTGTGCCA	ATCACTAACGGAGCAGTGAGC
capn1	NM_213459.1	CTCAGTTTATCGTGGATGGAG	CAGCAGATTATCATTCAGAGTC
casp3a	NM_131877.3	TCTACAATGACCAGACAGTTG	AAGACTTGAGATCCACAGATG
cyc1	NM_001037393.2	TGTGGTCGTGTTCTCCGGTA	AATGACATACTGGCCCGTGG
ccnd1	NM_131025.4	AAGTGGCTGCCTGTCTTGTT	ACTGCTGATTGTAGCTGCGT
ccne1	NM_130995.1	GCTTGGCTCAACATCTATATG	CAGACTCCTTACATCCAGAAT
ccng2	NM_213172.1	CGTGCCCGCAGACTCTAATA	AGGTTTCCAGCTAGGGAGGT
casp9	NM_001007404.2	CACGATTAGTACCACTGAGAC	CATCCATCTTGTAGCACTGTA
cdkn1a	NM_001128420.1	ATCCTACGTTCACTCGGTAAT	CCCTCTAGTGGTTTCTCAGAC
col1a2	NM_182968.2	TCTTGTTGGAGCCAGAGGTC	TGGGACCAGGGAATCCAATG
col1a1a	NM_199214.1	GAGGATGGTTGTACGTCGCA	AGGTGCACCAACGTCCATAG
col1a1b	NM_199214.1	CGACAAGGATGTATGGAAACC	GATTGGCACAGTCTGATGTATC
col2a1b	NM_001281478.1	AAGAACTGGTGGACTACGAAG	GAGGAAGGTCATCTGAATACTG
elavl1a	NM_131452.1	GAGACGTTGAAGTCGGAGGTG	CTGTGGCAGGTAGTTGACGA
fbxo25	NM_205724.1	GGCTGGAAGCGCTTTGATTA	TGGCAGCTACTTCACAGACA
fbxo32	NM_200917.1	CACAGACAGACAGATCCGCAA	GCAGTGCAAGGATGGTCTGT
foxO1a	NM_001077257.2	AGCGGCAAAGAAAAAGCTGG	CCGTCCAGGCTTCAAAGTCA
foxO3	NM_131085.1	GAGCAGTGATGAACAGGAA	TTGATGAGGTTGACGATGG
foxO4	XM_009291168.3	TCATACCCTCACAGGACCGT	ACCCTGTCCACCCTGTATGA
fn1a	NM_131520.2	GGAAGAGAATCAGGAGAGTGA	GAGAAGTCCAGGTGATAGTGA
gadd45bb	NM_001012386.2	GACATCGACATCAACATTGTG	CTCTTGGTTCATTGGCGTTAT
gnaq	NM_001144799.1	TCCCGAATATGATGGACCTCAG	TTTCCGTGTCAGTGGCACAT
gapdh	NM_001115114.1	ATCATCTCTGCCCCAAGTGC	ACGGTCTTCTGTGTTGCTGT
hif3a	NM_200405.1	TGGCTGCTAGATGGGACAAG	CCATCAGACAAGCCATCCAGT
itga11a	NM_001172627.1	AGGCCGGTCAGACATATAATCC	AACCCCAACTTCCAGAGTGC
igf1	NM_001172627.1	CATTGCCCGCATCTCATCCT	GACATCACACCAATGCCCCT
igf1ra	NM_152968.1	CTTACGCACACATGAACGGC	CAAAGGGAGGAGGGAAATGTT
lc3a	NM_214739.1	CCATCCGACAGACCCTTCAA	ATACCTCTCAATGATCACCGGAA
mdm2	NM_001365432.1	TGCAAACAGCAACTCGGATG	ACTCCACCTCAAACTCCACAC
mcm6	NM_001082849.1	GTCAAGGAGGCTTTCAGGCT	AGGTACATCGTGCCCATTCA
mcm3	NM_212567.2	TTCTTGACGACGACCAAGACC	GCTTTGCTGCTCGCTTTTCA
nd4	NP_059340.1	CCAACCTAGCACTTCCACCT	TCTGGGATTGAGCCTCGTTG
nd5	NP_059341.1	TCGCCACATCATTTACCGCA	TCAGGCAAGCCGTTGAATAGT
ppargc1a	XM_017357139.2	GCGAGGGAACGAGTGGATTT	CTCTCCACACCGAATCCTGA
pmaip1	NM_001045474.3	AAAGAGCAAACCGCTGTAGTA	TTCTGGAGTGTTACTATGAGC
prkaa1	NM_001110286.1	TCAACTGCTTGTACAGTCGCA	TCATAATGCGGCGGTTGTCT
pik3r4	XM_005158299.4	AAGAGAGCTCGCAAACATACG	TCCACTGTCTGGAATCGTCG
pik3r3b	NM_201143.2	ACAGCATGTCGGTATGTCAGTT	GGGAGCTGGCTAACTCTAACA
pik3r2	NM_212822.2	CAGCCTCCTTGCAGGTTGTT	CTCGGTCGCTGATTGTCAGA
rac3b	NM_001320406.1	AACGTCCGTGCAAAGTGGTA	GTGGGTAAGTGATGGGCGAT
sqstm1	NM_001312913.1	GGCTGGAACCCGTTACAAGT	CCTGAGCTTGAGCATTTGCC
trim63a	NM_001002133.1	ATTCTCCCGTGCCAACACAA	CGAAGCGACAAGTAGGGCAT
trim63b	NM_201095.1	CTTCAGAGGAATCTTCTTGTTG	GAGTAACGCAGTAGATGTTGA

tp53	NM_001271820.1	GCAGCGATGAGGAGATCTTT	GGGCTCAGATGATTCACGAT
tsc1b	NM_001282392.1	AGCTTACTGCCAAGTTGTCCT	TATCCGGTCCAGCCTGATGA
tsc2	NM_001328401.1	GTTTGACGACACACACGAGC	TTGGTCGTCCGCAAACTCTT
tnc	XM_021475994.1	AAGTCCTCGATTCAAGCGGC	TTCGGTGTTCCAAGCCCTGT
tuba1c	NM_001105126.2	TTCATCCTCCACACAGACTGC	GGATAGAGATGCACTCACGCATT
vwf	NM_001281989.1	GAAGCACATACTCTCATTCAG	GAACACTTCCTCATTCACTTG
