# Supplementary figures and images for: Identification of Potentially Related Genes and Mechanisms Involved in Skeletal Muscle Atrophy Induced by Excessive Exercise in Zebrafish
Source: Biology (Basel). 2021 Aug 10;10(8):761. doi: 10.3390/biology10080761 (PMC8389602; doi:10.3390/biology10080761)

# Western blot original picture

Figure S1F and S1H

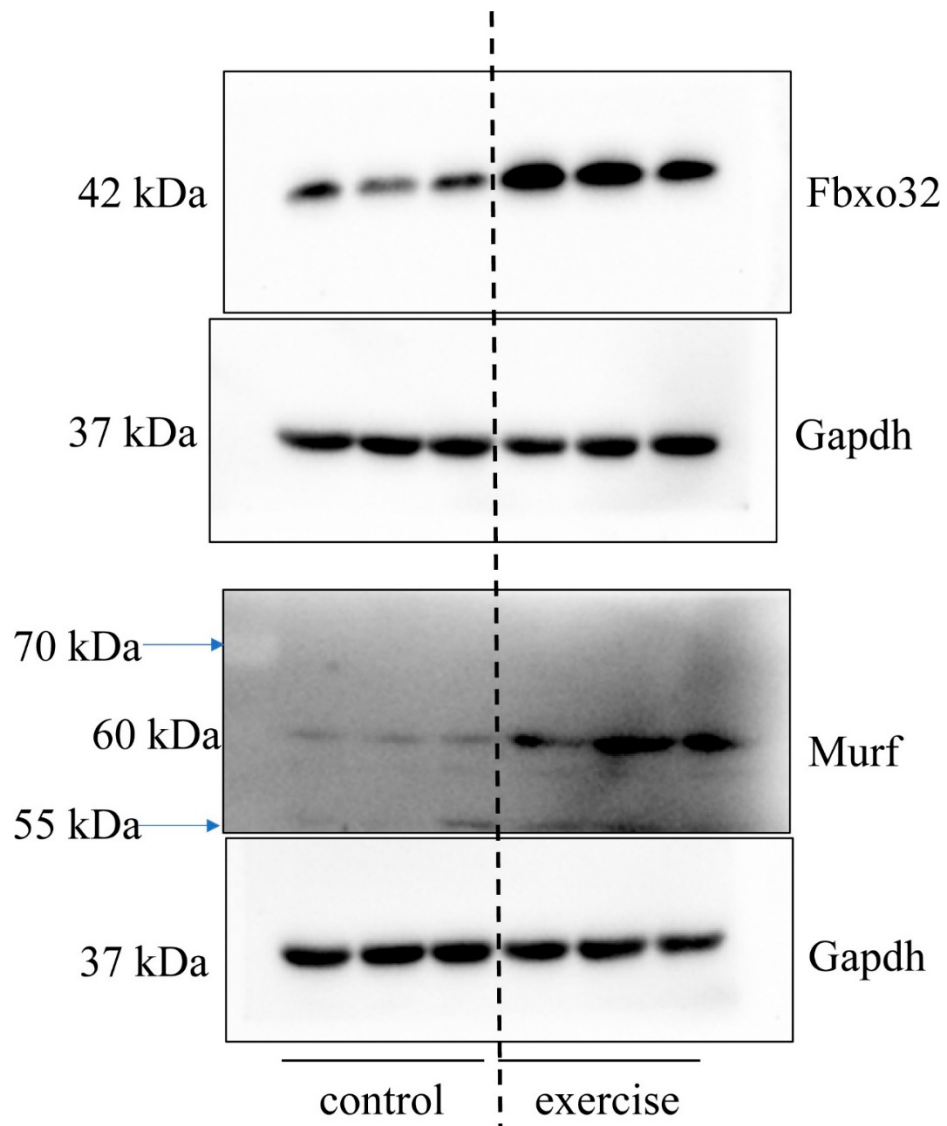

Figure S5A

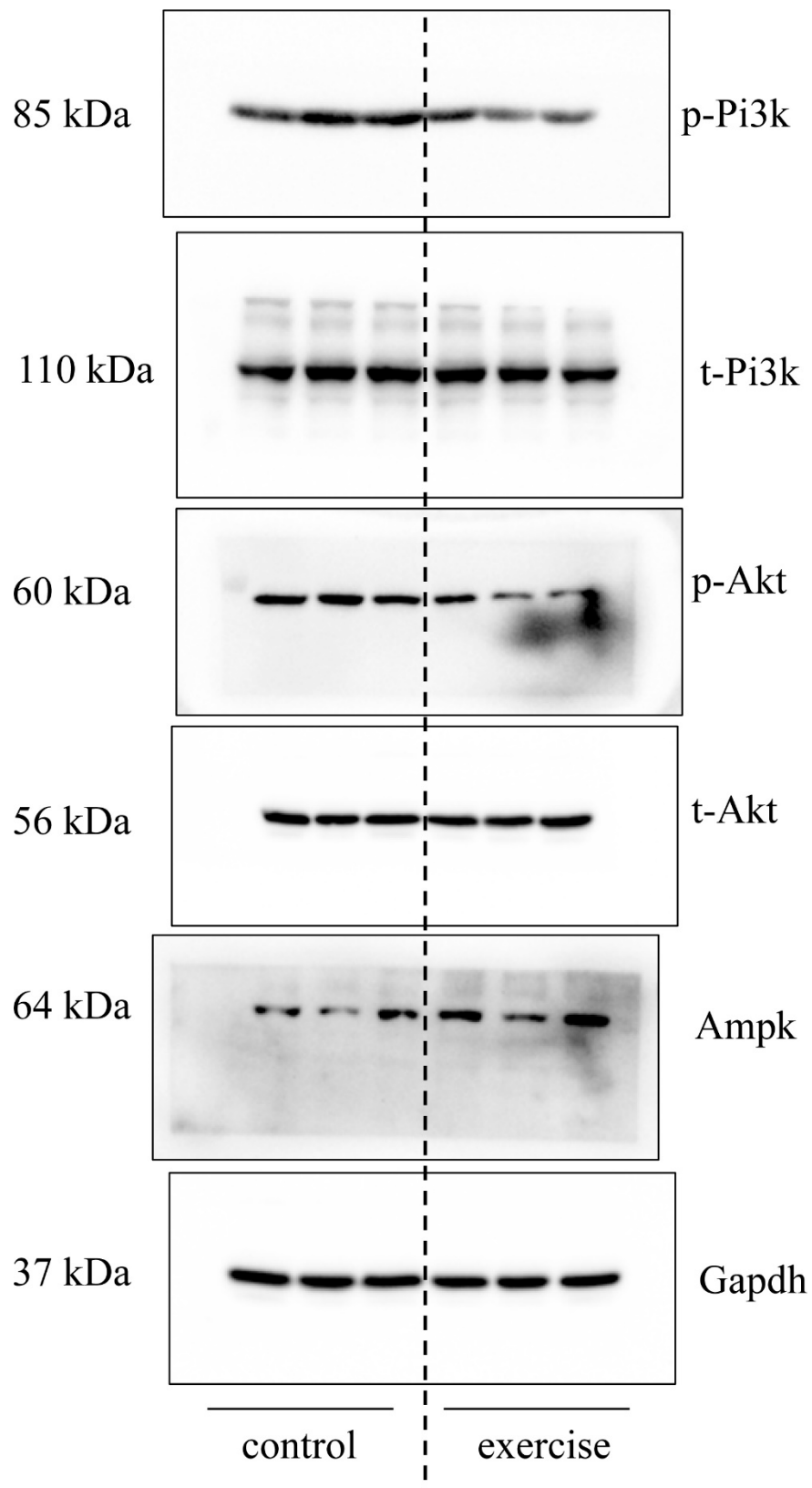

Figure S5B

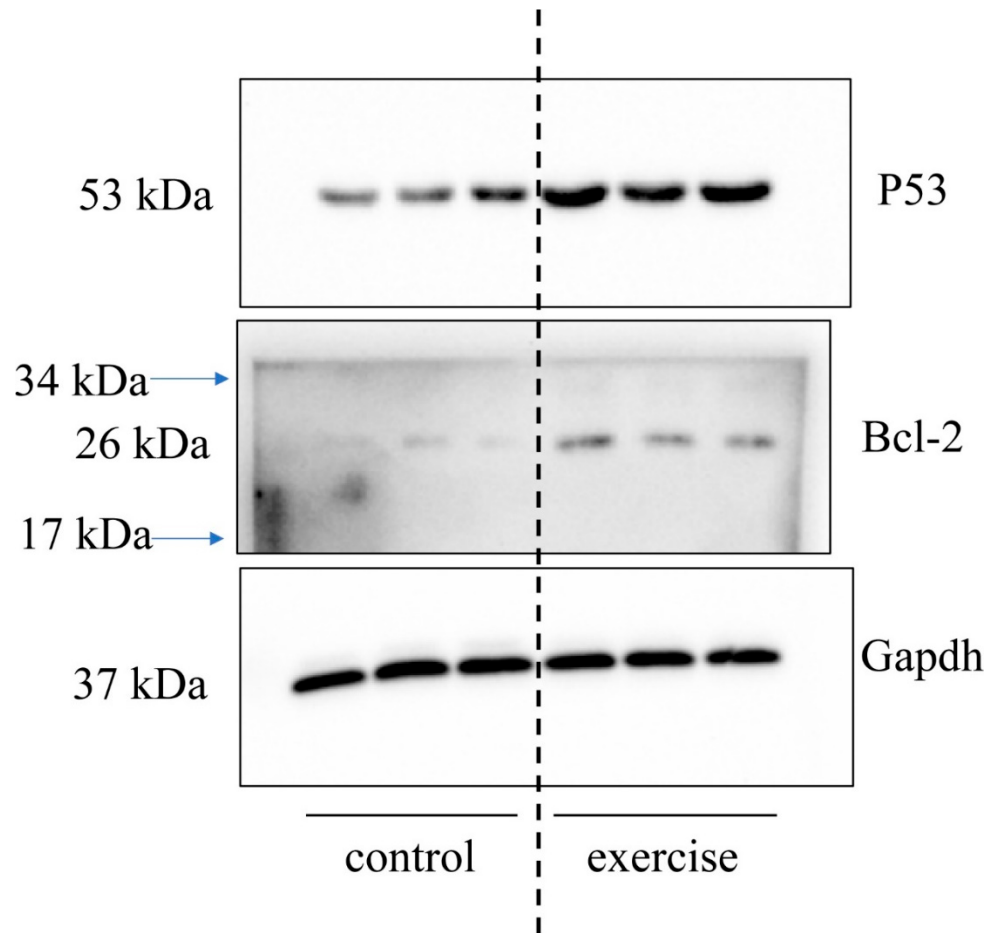

Figure S5C

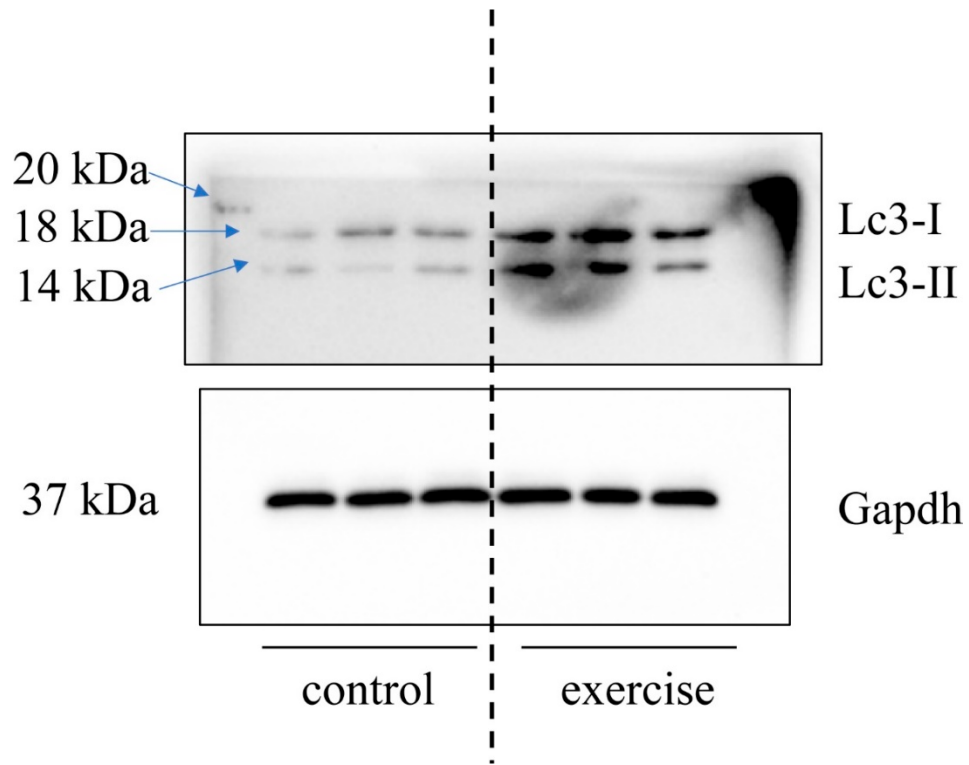

Supplement: Supplementary file 1 [file biology-10-00761-s001.zip › Western blot original picture.pdf]
